# Rapid Classification and Recognition Method of the Species and Chemotypes of Essential Oils by ATR-FTIR Spectroscopy Coupled with Chemometrics

**DOI:** 10.3390/molecules27175618

**Published:** 2022-08-31

**Authors:** Eleonora Truzzi, Caterina Durante, Davide Bertelli, Benedetta Catellani, Samuele Pellacani, Stefania Benvenuti

**Affiliations:** 1 Department of Life Sciences, University of Modena and Reggio Emilia, Via G. Campi 103, 41125 Modena, Italy; 2 Department of Chemical and Geological Sciences, University of Modena and Reggio Emilia, Via G. Campi 103, 41125 Modena, Italy

**Keywords:** partial least squares discriminant analysis (PLS-DA), principal component analysis (PCA), multivariate statistical analyses, chemotypes, attenuated total reflectance-Fourier transform infrared spectroscopy

## Abstract

In the present work, the applicability of attenuated total reflectance-Fourier transform infrared (ATR-FTIR) spectroscopy, coupled with chemometric tools in recognizing essential oils (EOs) for routine control, was evaluated. EOs belonging to *Mentha*, *Cymbopogon*, and *Lavandula* families and to *S. rosmarinus* and *T. vulgaris* species were analyzed, and the performance of several untargeted approaches, based on the synergistic combination of ATR-FTIR and Partial Least Squares Discriminant Analysis (PLS-DA), was tested to classify the species and chemotypes. Different spectra pre-processing methods were employed, and the robustness of the built models was tested by means of a Receiver Operating Characteristic (ROC) curve and random permutations test. The application of these approaches revealed fruitful results in terms of sensitivity and specificity, highlighting the potentiality of ATR-FTIR and chemometrics techniques to be used as a sensitive, cost-effective, and rapid tool to differentiate EO samples according to their species and chemotype.

## 1. Introduction

Essential oils (EOs) are complex mixtures extracted from aromatic plants by steam distillation or cold pressing. EOs are composed of mono and sesquiterpenes, and their concentrations may extremely vary depending on the type and pedoclimatic conditions of growth of the aromatic plant from where they are extracted [1]. Nowadays, EOs are commonly employed in the food, cosmetic, pharmaceutical, and perfume industries. Moreover, due to their strong antioxidant, antimicrobial, and pesticide effects, EOs have been intensively studied to increase the food shelf-life and the yields of crops in agriculture [2,3,4]. Thus, further applications of EOs, at the industrial level, are not an unrealistic scenario.

The EOs of the same genus, and in some cases, also from the same species, might largely vary in the chemical composition. In the latter case, the growth of aromatic plants, such as *Salvia rosmarinus* and *Thymus vulgaris*, is extremely affected by the pedoclimatic conditions, leading to different chemotypes of EOs [5,6]. The identity and the composition of the EOs are important factors from different points of view: safety, efficacy, and economy. The chemical characterization of the EOs is necessary at the industrial level to assure the quality of the final product and then, to safeguard the consumers’ health [7]. Moreover, an analytical controlled raw material employed in the manufacturing of foods, supplements, and personal care products guarantees the obtainment of a reproducible end-product in terms of efficacy and flavor. Indeed, the larger variations in the composition of raw materials can be compensated by process adjustments during the manufacturing of the end products [8]. Finally, the economic aspect concerns not only the industries but also the small-scale producers of EOs. Indeed, small farms do not invest in the characterization of their EOs due to the high costs of the analysis, resulting in the devaluation of their products. For this reason, in commerce, several EOs are sold without any declaration of the chemotype. Currently, gas chromatography (GC) is the most accurate and precise method to classify the EOs in terms of origins and composition [7].

However, GC is an expensive and time-consuming method that needs adequate infrastructure related to the use of inflammable and explosive gases. Therefore, the development of new precise, accurate, and rapid analytical methods is required for quickly classifying and evaluating EOs for quality assurance and control. Recently, Rasekh et al., validated a new method for the classification and identification of EOs with the employment of the electronic-nose technology [9]. Conversely, this approach might not be suitable in cases of dilutions of the EOs with non-volatile substances that cannot be detected by the electronic nose. The attenuated total reflectance-Fourier transform infrared (ATR-FTIR) spectroscopy showed to be an efficient tool in the quality control of the EOs. Indeed, recent reports highlighted the capability of this analytical method in determining adulterants [10,11,12,13] or classifying the EOs based on the plant family [14]. ATR-FTIR spectroscopy is a highly reproducible, sensitive, cost-effective, and rapid technique that does not require any sample preparation or specific infrastructures. The ATR mode is founded on the total internal reflection mechanism, which is useful to avoid the saturation of signals. Moreover, in multiple-reflection ATR, since the beam is reflected towards the samples several times, the examination of weakly absorbing samples can be achieved [15]. For these reasons, ATR-FTIR spectrometers are commonly used instruments at the industrial level. Schulz and co-workers demonstrated that ATR-FTIR spectroscopy is a suitable technology for the quantification of the major compounds of several EOs by employing multivariate statistical analysis of regression [16,17]. Multivariate statistical analysis might also be employed for the supervised classification of the EOs, depending on their infrared spectra. In particular, the partial least squares discriminant analysis (PLS-DA) is a powerful tool able to classify different samples depending on the variance of certain variables that characterize them [18]. The creation of an extended library of EO spectra and their analysis by PLS-DA might guarantee the rapid control of the EOs. Thus, information regarding the identity, main composition, and quality of the EOs can be obtained in a few minutes. In the present work, principal component analysis and PLS-DA were applied to ATR-FTIR data in order to test the applicability and the efficacy of the ATR-FTIR spectroscopy in recognizing and classifying the species and chemotypes of several investigated EOs. In particular, EOs belonging to *Mentha*, *Lavandula*, and *Cymbopogon genus* and *Salvia Rosmarinus* and *Thymus vulgaris* species were considered for the study, due to their economic importance and large or emerging use in the food industry. Moreover, these EOs were chosen for their variable chemical composition, characterized by high contents of monoterpenes belonging to different classes of organic compounds (aldehydes, ketones, alcohols, esters, alkenes). First of all, PCA was carried out as an explorative analysis, in order to obtain an overview on the whole data set, without forcing any model and to extract relevant information. PLS-DA was used to create classification models based on ATR-FTIR spectra. Different preprocessed spectra methods were tested, and the predictive ability of the constructed models was assessed by means of internal (cross-validation, CV) and external prediction.

## 2. Results and Discussion

In the present work, the raw spectra were employed for the following statistical analyses without any modification. Notwithstanding that ATR spectroscopy induces shifts in the vibrational signals compared to transmission spectroscopy, ATR correction was not performed since all the spectra were acquired in the same manner.

### 2.1. Characterization of the EOs by GC and ATR-FTIR Analyses

#### 2.1.1. Mentha Genus

The most available EOs on the market, of the Mentha genus, are obtained from the species arvensis (MA), piperita (MP), and spicata (MS). As can be seen in Table S1, MA and MP EOs exhibited similar compositions. Both the EOs were rich in the alcoholic monoterpene menthol, the ketonic monoterpenes menthone and isomenthone, and the ester methyl acetate, accordingly to the literature [19,20,21,22]. On the contrary, the composition of MS-EOs extremely differed from the other two species. Concerning the concentration, MS-EO was mainly rich in carvone and limonene, which accounted for more than 80% of the total composition. In addition, concentrations higher than 1% were detected for β-pinene, myrcene, iso-menthone, and α-terpineol [23]. Interestingly, two different groups of MA could be observed in terms of composition. Indeed, six out samples of MA-EOs showed a low content of menthol (35–54%) and high content of both menthone and its structural isomer (around 22% and 15%, respectively) [24]. These EOs demonstrated a chemical composition almost equal to MP-EOs. The main differences within these samples relied on minor monoterpenes, such as isopulegol and carvone (higher in MA), as well as limonene, 1,8-cineole, menthyl acetate, and β-caryophyllene (higher in MP). Conversely, the other MA-EOs displayed opposite abundances, where menthol ranged from 65 to 82%, and the ketones were, in all the cases, lower than 10%. To differentiate these two types of chemotype of MA, the latter case will be classified as the “menthol rich” chemotype.

**Figure 1:**
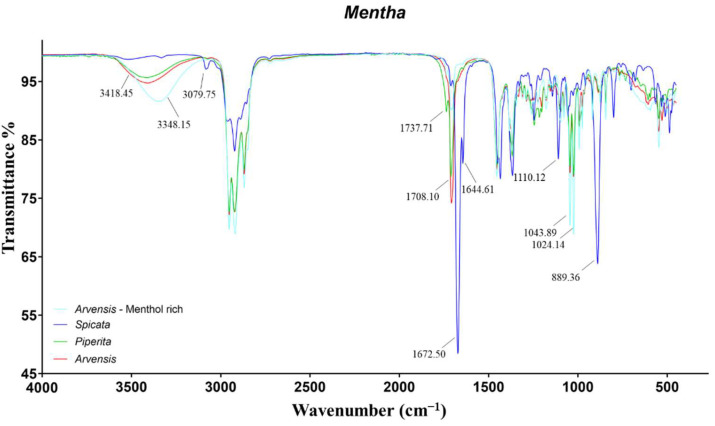
ATR-FTIR spectra of *Mentha piperita*, *Mentha spicata*, and two *Mentha arvensis* with different content of menthol.

The differences in the composition within the three species could also be observed in the acquired ATR-FTIR spectra (Figure 1). The assignments of the spectral bands have been performed in agreement with the literature [10,25].

The spectrum of MS-EO appeared extremely different from the others, with several peaks related to functional groups that were not detected in the other spectra. The signals at 3079 and 889 cm^−1^ were induced by C=C stretch and C−H out-of-plane bend of terminal vinyl groups, respectively, characteristic of limonene, myrcene, sabinene, and carvone. The intense peaks at 1672 cm^−1^ and 1644 cm^−1^ were assigned to the conjugated carbonylic group of carvone. Specifically, the first frequency was related to the C=O stretch, while the second was to the C=C stretch of the conjugated double bond. Finally, the last distinct peak of MS-EO might represent the C−O stretch of the tertiary alcohol α-terpineol at 1110 cm^−1^.

The spectra of both the chemotypes of MA and MP-EO showed the same signals with variable intensity, induced by the different abundance of the same monoterpenes. The peaks at 3418 and 3348 cm^−1^ represented the O−H stretch of the hydroxyl group of menthol. In the case of menthol-rich MA, the band was shifted at lower frequencies, due to the higher abundance of the alcoholic monoterpene, leading to stronger intermolecular hydrogen bonding [11]. Furthermore, the high concentration of menthol caused more intense signals at 1043 and 1024 cm^−1^ concerning the C−O stretch of secondary alcohols. Finally, the peak at 1708 cm^−1^ was induced by the C=O stretch of menthone and iso-menthone. The only characteristic band of MP-EO that was not evident in MA-EO concerned the C=O stretch of menthyl acetate at 1737 cm^−1^.

The remaining bands, common in all the Mentha EOs, were related to symmetric and asymmetric C−H stretching, bending, and rocking of –CH, −CH_2_, and –CH_3_ of alkane chains.

#### 2.1.2. Salvia Rosmarinus

Several chemotypes have been reported in the literature, based on the analysis of the higher relative percentages of the abundance of certain components [5,26,27,28]. In most of the collected rosemary EOs, the chemotype was not reported on the label due to the lack of the assignment from the producer. Napoli et al., stated that three main chemotypes are present: cineoliferum (high content in 1,8-cineole), camphoriferum (camphor >20%), and verbenoniferum (verbenone >15%) [5]. Overall, the attribution of the chemotype was performed according to this criterium, setting the minimum acceptable percentage of 1,8-cineole to 45%. Verbenone chemotype was assigned to the EOs with high concentrations of both verbenone and bornyl acetate. Finally, those EOs that did not fulfill these criteria and showed high percentages of α-pinene were classified as pinene chemotype. Representative chemical compositions of the four chemotypes identified among the rosemary EOs are displayed in Table S2.

**Figure 2:**
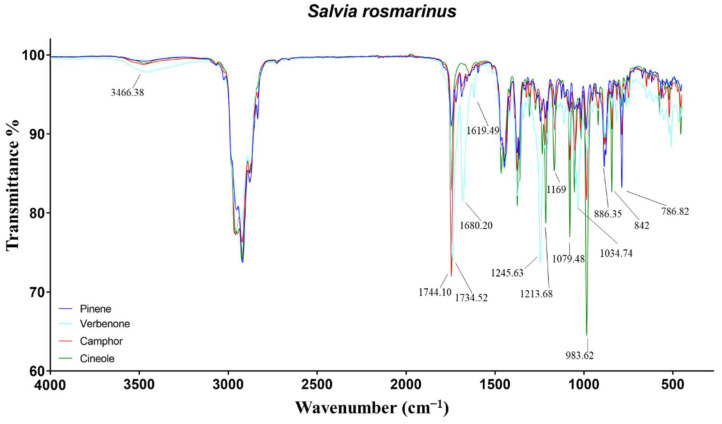
ATR-FTIR spectra of pinene, verbenone, camphor, and cineole chemotypes of *Salvia rosmarinus*.

The different composition of the four chemotypes was reflected in well-distinct IR spectra (Figure 2), due to the variable intensity of the same peaks or the presence of characteristic signals. The spectrum of pinene EO resulted as almost superimposable to both cineole and camphor EOs, due to the lack of relevant functional groups. Indeed, the monoterpene pinene exhibits one double bond, and its signals might be hidden due to overlapping. The only distinctive bands for their intensity were recorded at 886 and 786 cm^−1^, due to C−H out-of-plane bends [29].

In cineole-EO, the key peaks were attributed to the ether group of the monoterpene 1,8-cineole. Specifically, the signals at 1214 and 1079 were representative of C−O−C asymmetrical and symmetrical stretching, respectively. Furthermore, the wagging vibration of CH_2_ in the monoterpene induced the most intense band in the spectra at 983 cm^−1^ [30]. The peak at 1167 cm^−1^, particularly marked in cineole-EO, was not identified. Regarding camphor-EO, several peaks in the spectrum were found overlapped with those of cineole-EO but with a minor intensity. The only noteworthy signal was the C=O stretch of the ketone camphor at 1744 cm^−1^ [31]. Finally, verbenone-EO has been demonstrated to be the most divergent among the rosemary EOs, due to high concentrations of monoterpenes almost absent in the other EOs. The O−H and C−O stretching of borneol were recorded at 3466 and 1034 cm^−1^, while the carbonyl stretch of its ester was not identified, probably due to the presence of camphor stretching at 1734 cm^−1^. On the contrary, the C−O stretch of bornyl acetate at 1245 cm^−1^ stood out for its intensity. Regarding verbenone, the signals at 1680 and 1619 cm^−1^ were attributed to the C=O and C=C stretch of the conjugated carbonyl [25].

#### 2.1.3. Cymbopogon Genus

The *Cymbopogon* genus comprises about 180 species, subspecies, varieties, and subvarieties. However, the most important commercial species for the production of EOs are *C. martinii* (CM), *C. citratus* (CC), *C. nardus* (CN), and *C. winterianus* (CW). These EOs are largely employed in the food, pharmaceutical, cosmetic, and perfume industries as excipients. The chemical composition of the four species analyzed is reported in Table S3.

The CM-EOs (also called palmarosa EOs) were mainly composed by the alcoholic monoterpene geraniol, which ranged between 78 and 88%. In addition, noteworthy concentrations of geranyl acetate and linalool were detected [32,33]. The EOs of CC (also called lemongrass) exhibited a high abundance of neral, geranial, and geraniol that accounted for 80% of the total. The high variability of the concentrations of mono and sesquiterpenes is probably due to different growing locations [34,35]. The EOs belonging to CW and CN species (generally named as citronella or java EOs) showed a similar composition, with high contents of citronellal, geraniol, citronellol, and limonene. Their composition was in agreement with the observations discussed by several authors [11,36,37], and their high similarity is due to the fact that *C. winterianus* species was originated from *C. nardus* by clonal selection and mutation. The chemical composition of CW and CN EOs was observed to extremely vary within the samples of the same group. Indeed, some EOs exhibited lower content of both citronellol and citronellal, as well as higher content of geraniol and/or camphene. The great variability in the composition might be explained by the fact that these plants can be widely affected by the pedoclimatic conditions of growth [38,39,40].

**Figure 3:**
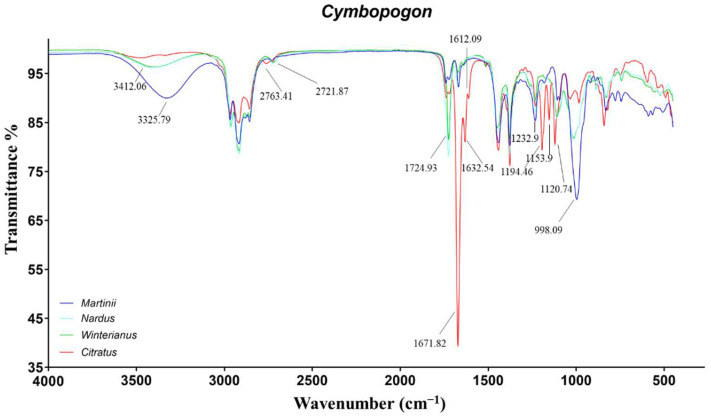
ATR-FTIR spectra of Cymbopogon martinii, Cymbopogon nardus, Cymbopogon winterianus, and Cymbopogon citratus.

Regarding the ATR-IR spectra, great differences within palmarosa, lemongrass, and java EOs were highlighted. On the contrary, as expected by observing the GC results, important dissimilarities between CW and CN were not identified (Figure 3). The main differences were related to the different ratios of the alcoholic monoterpenes, geraniol and citronellol, and of the aldehydes: citronellal, neral, and geranial.

The spectra of CM-EOs were mainly characterized by the signals of geraniol. In particular, the peaks at 3325, 1669, 1233, and 998 cm^−1^ were induced by the primary O−H stretch, C=C stretch, C−C stretch and C−O stretch, respectively [41]. Additionally, the small signal at 1728 cm^−1^ was assigned to the carbonylic stretch of geranyl acetate. The main signals in the spectra of CC-EOs were linked to the conjugated aldehydes, neral and geranial. Specifically, the peaks at 2763, 1671, 1632 (and 1612) cm^−1^ were related to aldehydic C−H stretch, conjugated C=O stretch, and conjugated C=C stretch, respectively [42]. The further signals at 1194, 1154, and 1120 cm^−1^ were tentatively assigned to the conjugated aldehyde C−C stretch.

Finally, the spectra of CW and CN-EOs exhibited the characteristic signals of primary alcohols at 3412, 1233, and 998 cm^−1^, induced by citronellol and geraniol. Moreover, the saturated aldehyde citronellal caused the vibrations at 2722 and 1725 cm^−1^, due to the aldehydic C−H and C=O stretches [11,25].

#### 2.1.4. Lavandula Genus

*Lavandula genus* is mainly represented by the most diffuse species *L. angustifolia* (LA), *L. x intermedia* (LI), and *L. latifolia* (LL). The composition of LA and LL-EOs were shown to differ in the content of several mono and sesquiterpenes, such as 1,8-cineole, linalool, camphor, and linalyl acetate. On the contrary, LI-EOs, *L. x intermedia* being a hybrid plant between *L. angustifolia* and *L. latifolia*, displayed an intermediate composition (Table S4). In all these species, linalool was the most important monoterpene, representing 30–40% of the whole oil composition. Then, LA and LI-EOs contained high concentrations of linalyl acetate, terpinene-4-ol, lavandulyl acetate, and ocimene, which were present, in traces, in LL-EOs. On the opposite, LL-EOs showed a great abundance of 1,8-cineole and camphor among the other terpenes. The compositions were in agreement with the outcomes of other authors in the literature [43,44,45].

**Figure 4:**
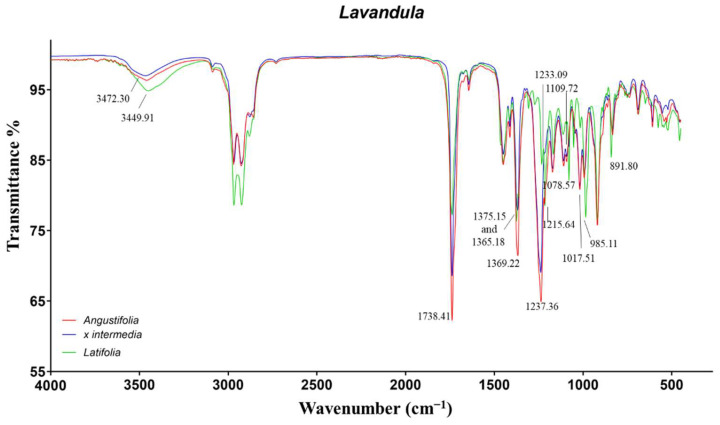
ATR-FTIR spectra of Lavandula angustifolia, Lavandula x intermedia, and Lavandula latifolia.

The similar composition of the EOs was reflected into the ATR-FTIR spectra (Figure 4). Indeed, the majority of IR vibrations were in common for all the species with different intensities. The most representative signals were related to linalool, linalyl acetate, camphor, and 1,8-cineole.

The LL-EO differed from the other two species for the characteristic signals of 1,8-cineole also detected in rosemary EOs. In particular, the vibrations at 1215, 1078, and 985 cm^−1^ were induced by C−O−C asymmetric and symmetric stretch, as well as wagging vibration of CH_2_, respectively. The peak at 891 cm^−1^, also observed in the cineole chemotype of rosemary EOs, was not identified. The remaining signals were in common with the LA and LI-EOs. The tertiary alcohol linalool induced the O−H broad stretch and bend in the range 3472–3449 cm^−1^ and 1375–1365 cm^−1^, respectively. The signal at 1369 cm^−1^ was more intense for LA-EO, probably due to the contribution of several secondary and tertiary alcoholic monoterpenes. The signals produced by the carbonylic groups of camphor and linalyl acetate resulted, superimposed, at 1738 cm^−1^. Additionally, linalyl acetate exhibited a further characteristic peak at 1237 cm^−1^, induced by the ester C−O stretch. Finally, the signals around 1109 cm^−1^, also observed in our previous work [11], were not identified.

#### 2.1.5. Thymus Vulgaris

Thyme EOs present in the market are extremely variable, exhibiting several chemotypes, as with rosemary EOs. In the literature, 13 different chemotypes of *T. vulgaris* have been reported depending on the predominance of a singular monoterpene [6]. The majority of EOs collected from local producers or sellers did not report the type of chemotype on the label. Furthermore, in some cases the declared chemotype was incorrect. For this reason, for all the EOs, the proper classification was assigned after the semi-quantitative analysis in GC. In Table S5, the typical chemical compositions of the main chemotypes of *T. vulgaris* EOs that were encountered are displayed. In addition to these, some samples exhibited a multi-component chemotype, being rich in more than one type of monoterpene [46]. Among these chemotypes, thymol-EOs usually displayed, in addition to thymol (> 30%), high abundances of *p*-cymene and γ-terpinene [47]. Borneol-EOs resulted in being mainly represented by borneol, camphene, α-terpineol, and α-pinene. Finally, linalool-EOs demonstrated high concentration of alcoholic monoterpenes, such as linalool and terpinene-4-ol, in addition to aliphatic hydrocarbon terpenes (α-pinene, cymene, and α-terpinene) [6].

The EOs that exhibited high contents of *p*-cymene and lower contents of the main terpenes above-identified were classified as the cymene-thymol chemotype. The EOs that showed more than 2 representative terpenes were classified as multicomponent.

**Figure 5:**
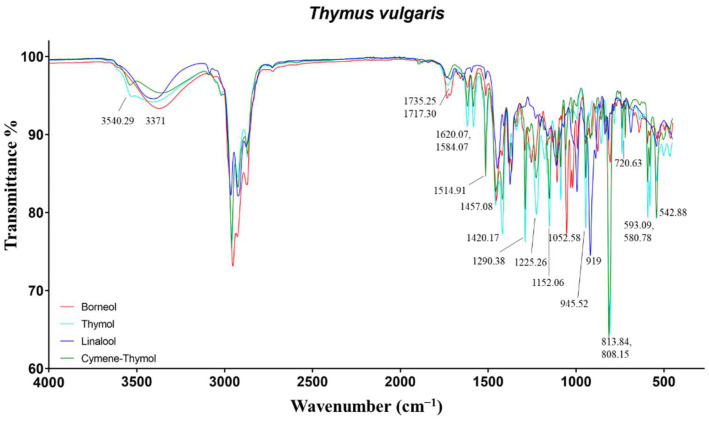
ATR-FTIR spectra of borneol, Thymol, Linalool, and Cymene-Thymol chemotypes of *Thymus vulgaris*.

The spectra of these EOs reflected the differences observed in the compositional results (Figure 5). As expected, thymol and cymene-thymol-EOs differed in the intensity of the peaks related to *p*-cymene and thymol. The most important vibrations of thymol, present at 3540, 1620/1584, and 1290 cm^−1^, were assigned to the O−H stretch, aromatic C=C−C ring stretch, and phenolic C−O stretch. *p*-cymene, being an aromatic hydrocarbon, exhibited several signals in the fingerprint region at 1514, 813, 720/542 cm^−1^, due to aromatic C=C−C ring stretch, C−H of para disubstituted aromatic rings, and aromatic C−H out-of-plane bends. Several additional signals in the range 1458–1380, 1228–943, and 860–810 cm^−1^ were identified as aliphatic C−H asymmetrical/symmetrical bending, aromatic C−H in-plane bends, and aromatic C−H out-of-plane bends [16,25,48].

Borneol-EO mostly differed for the vibrations of the alcohols borneol, carvacrol, and α-terpineol, the ketones camphor and carvone, and the hydrocarbon monoterpene camphene. In particular, the alcohols induced the O−H stretch at 3371 cm^−1^, as well as the C−O stretch at 1052, 1031 (borneol, as for rosemary verbenone-EO), and 1019 cm^−1^. The C=O stretch of carbonylic groups of bornyl acetate and camphor were present at 1735 and 1717 cm^−1^, respectively. The hydrocarbon camphene exhibited the vibration at 876 cm^−1^ linked to vinylidene C−H out-of-plane bend.

Finally, linalool-EO showed the characteristic O−H stretch at 3402 cm^−1^, vinyl C−H stretch at 3088 cm^−1^, and vinyl C−H out-of-plane bends at 919 cm^−1^ related to alcohols and terminal double bonds, respectively.

### 2.2. Principal Component Analysis (PCA)

The PCA was applied to the whole data matrix (composed of absorbance spectral variables) to have a general overview of the trend, similarities, and differences among the investigated samples, according to their IR profiles. The PCA is commonly used to summarize the information included into datasets by extracting new artificial variables called principal components (PCs). These new variables intrinsically carry the information of the original variables, extrapolating the most important underlying information of the dataset. By projecting samples onto the first few PCs, the structure of the investigated data can be visualized into a lower-dimensional space. PCA is strongly recommended prior to supervised classification methods (e.g., PLS-DA), with it being an informative predictor to comprehend whether reliable PLS-DA models might be trained on the data [49].

Four principal components were considered according to their explained variances (R^2^: 81%).

**Figure 6:**
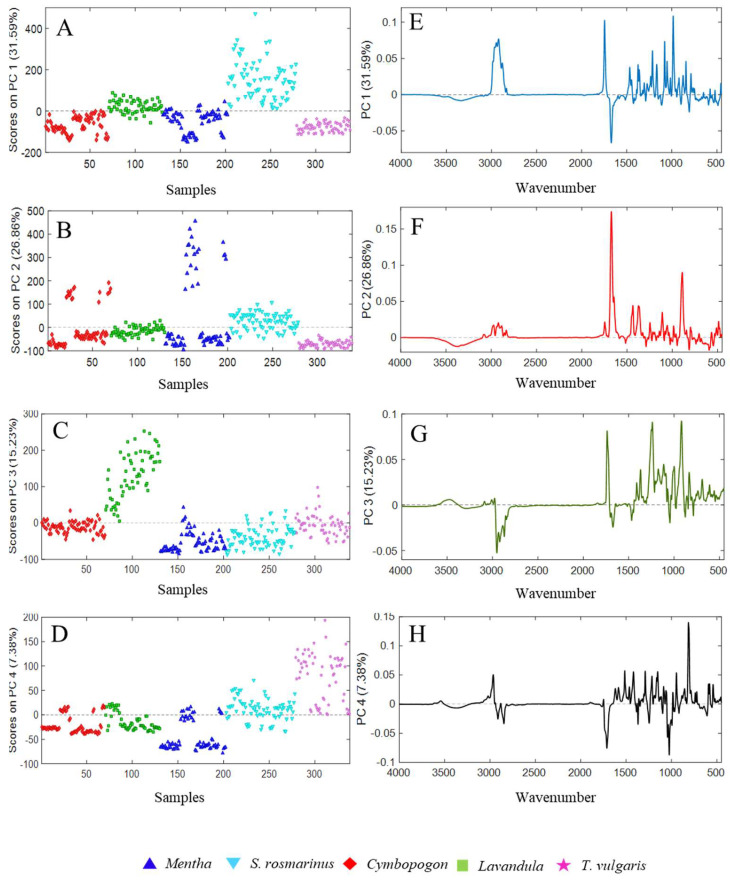
The scores on the extracted PCs ((**A**–**D**) for PC1, PC2, PC3, and PC4 respectively) and their loading plots ((**E**–**H**) of PC1, PC2, PC3, PC4, respectively), obtained from the principal component analysis (PCA) performed on the spectral data of the essential oils belonging to *Mentha*, *Cymbopogon*, and *Lavandula genus*, as well as *Salvia rosmarinus* and *Thymus vulgaris* species.

In Figure 6A–D, the score plots of PC1, PC2, PC3, and PC4 are reported. 

The different EOs are represented with different symbols and colors depending on their genus. Each component seems to slightly differentiate samples belonging to one class of EO. In particular, rosemary EOs got the highest scores for PC1, lavender for PC3, and almost all thymes for PC4. As far as PC2 is concerned, this component mainly highlighted differences within citronella and mint EOs. Indeed, only some EOs of these classes presented higher PC2 score values, showing different IR signals with respect to the other samples. Notwithstanding that the aim of the present study is to build a fast model able to extract information about the differences among the investigated EOs in an untargeted manner, some considerations on the presence of some chemical compounds can be done by looking at the loading plots of the selected PCs. From the loading plots of the PCs, displayed in Figure 6E–H, it is possible to point out that the separation among the different classes was mainly due to the spectral peaks between 3600–2600 nm and 1970–700 nm, which influence the trend of the scores in different ways. In particular, rosemary EOs exhibited the highest positive scores on PC1, followed by lavender EOs. These samples were mainly influenced by spectral bands that got the highest positive loadings on PC1at 1746 cm^−1^, related to unconjugated carbonyl groups (e.g., linalyl acetate and camphor), and at 1214, 1080, and 985 cm^−1^ induced by 1,8-cineole. Besides, lavender EOs were conditioned by the peaks at 1375–65 cm^−1^ of the tertiary alcohol linalool, and at 1466 cm^−1^ that was not previously identified. This latter signal might belong to frequencies of saturated aliphatic groups [25]. On the other hand, signals induced by conjugated carbonyls around 1670 cm^−1^ played a central role in collocating the remaining EOs in negative on PC1. Lavender EOs were predominantly clustered by their typical peaks at 1738, 1370, 1244–1239, and 917 cm^−1^ (linalyl acetate, linalool, and 1,8-cineole), which gained the highest positive loadings on PC3. Finally, thyme EOs were positively separated from the remaining EOs, due to the high loadings on PC4, attributed to bands at 1516, 1419, 1289, 945, and 813 cm^−1^ related to *p*-cymene and thymol, as well as 1154 cm^−1^ induced by borneol. Furthermore, the absence of characteristic signals in the regions at 1745–1678 and 1045–993 cm^−1^ influenced the scores of these EOs, being important variables with negative loadings on PC4.

On the contrary, Mentha and Cymbopogon EOs were not completely separated. These latter EOs were partially influenced, instead, by the elevated PC2 loadings in the range 1672–1632 cm^−1^ and at 889 cm^−1^ which induced a split of the classes. These vibrations corresponded to conjugated carbonyl groups belonging to ketones and aldehyde (carvone and neral/geranial) typical of MS and CC-EOs that were notably different from the EOs from the same genus. Additionally, the frequencies at 1435–1365 and 1114 cm^−1^, belonging to saturated aliphatic groups and α-terpineol (present in MS-EOs), had a moderate role in the separations of Mentha e Cymbopogon classes.

### 2.3. Partial Least Squares Discriminant Analysis (PLS-DA)

PLS-DA models were separately carried out on each of the EOs spectra data sets belonging to the *Mentha, Cymbopogon*, and *Lavandula genus*. As far as chemotypes of *Thymus vulgaris* is concerned, it was not possible to build a classification model for each genus, due to the low number of samples. However, for this genus, a preliminary PCA showed a good differentiation among the different chemotypes (Figure S1). A first attempt of classification was made for *Salvia Rosmarinus* chemotypes with a higher number of EOs: namely, cineole and camphor. The remaining EOs belonging to pinene and verbenone chemotypes were classified as “others”. The PCA performed on the whole *S. rosmarinus* dataset is displayed in Figure S2.

During the acquisition of ATR-FTIR spectra, different phenomena—namely, sample background and instrument performance—can influence spectral data quality, and pretreatment of data could be a critical and case-dependent issue in multivariate analyses. Therefore, in this study, the following five different data preprocessing methods were evaluated: mean centering (MC), standard normal variate (SNV) + MC, multiplicative scatter correction (MSC) + MC, Savitzky–Golay first derivative (1st der) + MC, and Savitzky–Golay second derivative (2nd der). With regards to the first and the second derivatives, second order and third order polynomials, with seven points in each window, were used, respectively. The obtained results of PLS-DA models, using different pretreatments (the number of latent variables, LVs, Sensitivity and Specificity in cross-validation, root mean squares in CV and prediction, area under the ROC curve in CV, and permutation test results obtained with 50 randomizations), are reported in Table 1, Table 2, Table 3 and Table 4.

In all the developed models, the permutation results were lower than 0.05, indicating that the randomly permuted models were significantly different at the 95% limit, highlighting the clear robustness of the obtained classification models. Furthermore, it is worth noting that almost all the models presented a high accuracy since all AUC values were higher than 0.92; indeed, the closer the value of the AUC is to 1, the better the classification model. However, slight differences emerged in terms of sensitivity (percentage of objects belonging to the class correctly accepted by the class models) and specificity (percentage of objects not belonging to the class correctly rejected by the class model) in both CV and prediction models.

As the choice of the most suitable pretreatments is concerned, through a synergistic comparison of all the reported parameters, mean centering and first derivatives (+ MC) seemed to be the most efficient pretreatment methods in this research. However, first derivatives + MC pretreatments could be chosen since the respective models seemed to be more parsimonious, selecting a lower number of latent variables. Although only the three classification models of *Salvia Rosmarinus* achieved 100% in sensitivity and specificity, all other models showed a good performance, pointing out the potentiality of ATR-FTIR to detect chemical distinction among the different species. By examining the variable importance in projection (VIP) score plots in depth (Figures S3–S6), it was evident that the variables that received the highest scores were the most important and characterful signals of each EO spectrum (fully described previously). The VIP score plots estimate the importance of each variable in the projection used in the PLS-DA model. In other words, the variables with the highest scores and above the significant threshold are distinguishing for the classification. Therefore, the lower sensitivity and specificity of some models can be attributable to the similar and, sometimes, almost equal chemical composition of certain EOs, as previously discussed on GC results. Indeed, MA and MP, CW and CN, and LA and LI EOs, which have a similar chemical composition and produced similar spectra, displayed the same important variables. This evidence suggests the proximity and possible overlap of different EO species, impairing their complete classification. It is noteworthy to highlight that the highest percentages of misclassified EOs were recorded for *C. nardus* and *C. winterianus*, which are plant aromatic species that are genetically correlated. The same also applies to *L. x intermedia*, whose model did not exceed the 88% of sensitivity. Thus, the correct classification of these EOs is a difficult task to be achieved through GC analyses.

Finally, to have a general overview of the performance of the PLS-DA-assisted untargeted ATR-FTIR approach, PLS-DA was performed on the whole data set simultaneously, considering all the data acquired on samples belonging to five classes: namely, *Mentha, Lavandula,* and *Cymbopogon genus,* as well as the *Thymus vulgaris* and *Salvia Rosmarinus* species.

For building the model, 218 samples were used for calibration and 120 for external validation, and, based on the previous results, the signals were pretreated with Savitzky–Golay first derivative (1st der) + mean centering. The number of samples belonging to each class, for training and external validation sets, was reported in Table 5.

The number of latent variables for PLS models was chosen by the smallest mean squared error obtained in CV (venetian blind with 10 data splits).

The best EOs classification model was built with six latent variables, and this model gave excellent results in terms of robustness and classification performance (Table 6).

In particular, the model showed excellent sensitivity in both CV and prediction models, with all the samples correctly classified in the respective belonging class. As far as specificity is concerned, all the EOs not belonging to the class were correctly rejected by the class model except for two samples belonging to the *Thymus vulgaris* species, classified as *Lavandula* in both CV and prediction models. This result might be due to the close chemical composition of certain thyme EOs displaying the linalool chemotype.

**Figure 7:**
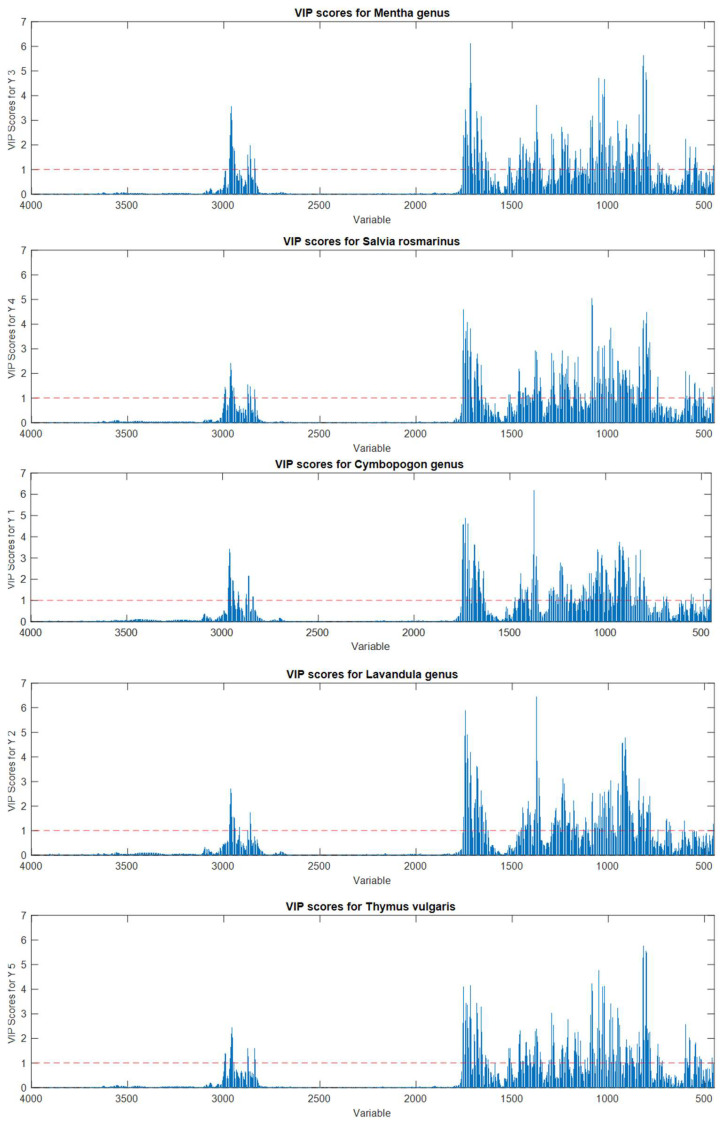
Variable importance on projection (VIP) score plots for the PLS-DA models for discriminating *Mentha*, *S. rosmarinus*, *Cymbopogon*, *Lavandula*, and *T. vulgaris* essential oils. Red dotted lines indicate the significance threshold.

The VIP score plots (Figure 7) showed that this differentiation is mainly due to regions between 3100 and 2500 cm^−1^ and 2000–500 cm^−1^. These regions were already highlighted as relevant by inspecting PCA loadings. The variables with the highest scores were strictly related to characteristic IR signals present in most of the EOs belonging to the same class, as expected. Indeed, for *Mentha* EOs, the most discriminating variables were related to ketones (carvone, menthone, and isomenthone, 1740–1640 cm^−1^), menthol vibrations (1100–1000 cm^−1^) and monoterpenes containing terminal vinyl groups (limonene, carvone, 880 cm^−1^). Similarly, *S. Rosmarinus* EOs were mostly represented by ketones (camphor and verbenone, 1750–1650 cm^−1^) and the heterocyclic monoterpene 1,8-cineole (1085 and 980 cm^−1^), while *Lavandula* EOs were represented by the stretch and bends of the carbonylic group of linalyl acetate (about 1740 and 1240 cm^−1^) and the hydroxyl group of linalool (1375–1365 cm^−1^). Cymbopogon EOs, being mainly composed of aldehydes and alcohols, were predominately projected by the variables around 1730–1600 and 1380 cm^−1^. Finally, concerning *T. vulgaris* EOs, the highest scores were reached by several variables in all the fingerprint regions, being the most variable in composition due to the expression of several different chemotypes. In particular, the signals belonging to alcohols (borneol, carvacrol, linalool, and α-terpineol), ketones (carvone and camphor), phenols (thymol), and hydrocarbons (*p*-cymene and camphene) contributed to the classification model.

The diversity of chemical composition and, then, functions of EOs belonging to the same genus or chemotype offers a great variety of applications. However, in some cases, certain compounds of EOs can also display features such as allergenicity or toxicity. Therefore, qualitative controls of EOs are certainly an important issue. The key technique for the identification of the type of EOs is the GC, where quantitative results of the chemical compounds allow the recognition by comparing the composition with reference monographs [7]. Alternative techniques proposed for the quality control of EOs are based on a fingerprinting approach, such as IR or nuclear magnetic resonance (NMR) spectroscopies. To the best of our knowledge, this is the first study present in the literature that aimed at the development of supervised classification models, of ATR-FTIR data, for identifying the species or chemotypes of EOs. In the literature, several examples of the application of fingerprinting methods for similar purposes are present. Baranska et al., demonstrated that vibrational spectroscopy, in combination with the unsupervised hierarchical cluster analysis, could discriminate eucalyptus EOs [30]. Besides, Lafhal and co-workers reported the great potentialities of near IR spectroscopy coupled with PLS-DA in discriminating lavender and lavandin EOs [50].

The creation of PLS-DA models for *Ocimum* EOs was attempted by Freitas et al., on NMR spectra and GC chromatograms, demonstrating the excellence of the NMR approach in recognizing the species [51]. Certainly, NMR is a powerful technique. However, to achieve the goal, ATR-FTIR spectroscopy displays several advantages. Indeed, ATR-FTIR spectrometers are extremely diffused, easy-to-use, and cheaper than NMR spectrometers. Moreover, the EO analysis does not require the employment of deuterated solvents and any sample preparation.

## 3. Materials and Methods

### 3.1. Materials

All the EOs were kindly donated by several companies fully cited in the acknowledgments. A total of 36 EOs belonging to the *Mentha arvensis* (MA), *Mentha spicata* (MS), and *Mentha piperita* (MP) were collected. On the label of all these samples, the species was specified by the producing/seller company. A total of 38 *Salvia rosmarinus* EOs was collected. The chemotype of the EO was not reported in the majority of the labels. A total of 35 EOs, belonging to *Cymbopogon winterianus* (CW), *Cymbopogon nardus* (CN), *Cymbopogon citratus* (CC), and *Cymbopogon martinii* (CM) were obtained. On the label of all these samples, the species was specified by the producing/seller company. Thirty EOs, belonging to *Lavandula latifolia* (LL), *Lavandula x intermedia* (LI), and *Lavandula angustifolia* (LA), were investigated. On the label of all these samples, the species was specified by the producing/seller company. Finally, a total of 32 *Thymus vulgaris* EOs were collected. The chemotype of the EO was not reported in the majority of the labels. *n*-Hexane and the mixture of aliphatic hydrocarbons (C_8_–C_40_) were purchased from Sigma-Aldrich (Milan, Italy).

### 3.2. Analysis of the EOs

#### 3.2.1. GC-MS Analysis

Analyses were performed on a 7890A gas chromatograph coupled with a 5975C network mass spectrometer (GC-MS) (Agilent Technologies, Milan, Italy). Compounds were separated on an Agilent Technologies HP-5 MS cross-linked poly−5% diphenyl–95% dimethyl polysiloxane (30 m × 0.25 mm i.d., 0.25 μm film thickness) capillary column. The column temperature was initially set at 45 °C, then increased at a rate of 2 °C/min up to 100 °C, then raised to 250 °C at a rate of 5 °C/min, and finally, it held for 5 min. The injection volume was 0.1 μL, with a split ratio 1:20. Helium was used as the carrier gas, at a flow rate of 0.7 mL/min. The injector, transfer line, and ion-source temperatures were 250, 280, and 230 °C, respectively. MS detection was performed with electron ionization at 70 eV, operating in the full-scan acquisition mode in the m/z range 40–400. The EOs were diluted 1:20 (v/v) with *n*-hexane before GC-MS analysis.

#### 3.2.2. GC-FID Analysis

Chromatographic characterization of EOs was performed on a 7820 gas chromatograph (Agilent Technologies, Milan, Italy) with a flame ionization detector (FID). EOs and the mixture of aliphatic hydrocarbons (C_8_–C_40_) were diluted 1:20 (v/v) with Hex before GC-FID analysis. Helium was used as the carrier gas at a flow rate of 1 mL/min. The injector and detector temperatures were set at 250 and 300 °C, respectively. EO components were separated on an Agilent Technologies HP-5 crosslinked poly−5% diphenyl–95% dimethylsiloxane (30 m × 0.32 mm, i.d., 0.25 mm film thickness) capillary column. The column temperature was initially set at 45 °C, then increased at a rate of 2 °C/min up to 100 °C, then raised to 250 °C at a rate of 5 °C/min, and finally, it maintained for 5 min. The injection volume was 1 μL, with a split ratio 1:20.

Compounds were identified by comparing the retention times of the chromatographic peaks, with those of authentic reference standards run under the same conditions, and by comparing the linear retention indices (*LRI*s) relative to C_8_–C_40_ *n*-alkanes obtained on the HP-5 column, under the above-mentioned conditions, with the literature [52]. Peak enrichment by co-injection with authentic reference compounds was also carried out. A comparison of the MS-fragmentation pattern of the target analytes with those of pure components was performed by using the National Institute of Standards and Technology (NIST version 2.0d, 2005) mass-spectral database.

The percentage relative amount of individual components was expressed as the percent peak area, relative to the total peak area obtained by the GC-FID analysis. Semi-quantitative data were acquired from the mean of two analyses.

The data acquisition and processing were performed using the OpenLab CDS C.01.04 (Agilent Technologies, Santa Clara, CA, USA) software.

### 3.3. ATR-FTIR Spectra Acquisition

The ATR-FTIR spectra of the EOs were obtained using a FT-IR spectrometer Spectrum Two, equipped with a Universal ATR sampling accessory (Perkin Elmer, Milano, Italy). One drop of each sample was deposited on the ATR diamond crystal cell. The spectra were acquired in the spectral range of 4000–450 cm^−1^, with a spectral resolution of 4 cm^−1^, averaging 16 scans per spectrum. The number of scans was selected for the optimal signal-to-noise ratio. The background spectrum of the empty ATR cell was obtained under the same instrumental conditions prior to the sample analysis. All samples were analyzed in duplicate.

For each EO, the absorbance percentages related to each wavenumber in the infrared range from 4000 to 450 cm^−1^ (3551 points in total) were exported to create the datasets.

### 3.4. Statistical Analysis

The multivariate statistical analyses were performed by using PLS_Toolbox 8.9.2 software (Eigenvector Research Inc., Manson, WA, USA) for MATLAB^®^. The PCA was performed on all the ATR-FTIR spectral data. All the IR signals were organized in a bidimensional matrix of dimension 338 × 3551 (i.e., essential oil samples × 3551 IR points) and pretreated by means of multiplicative scatter correction (MSC), followed by mean-centering. In this first step of exploratory analysis, MSC pretreatment was selected to minimize additive and multiplicative effects present in the baseline of the spectral data. The number of PCs was selected based on the best compromise between the smallest root mean squared error in leave one out cross-validation (RMSECV). A detailed investigation on the right choice of the signal pretreatment to be used was performed during PLS-DA analysis.

The PLS-DA can be defined as a multivariate technique that allows the creation of discrimination models, giving the maximum covariance between measured data (ATR-FTIR spectral intensities) and the response variable (represented, in this case, by the classification of samples based on the EOs species information) [53]. For building each PLS-DA model, the dataset was split into training and test sets, where at least 60% of the data set was selected for the calibration set according to the Kennard–Stone algorithm [54], being careful to insert the same replicates together. After each signal pretreatment, the right number of latent variables for each PLS-DA model was selected according to the best compromise between the smallest RMSECV in leave one out cross-validation.

Before building a classification model for Mentha, Lavandula, and Cymbopogon genus, as well as Thymus vulgaris and Salvia Rosmarinus species, the possible presence of outliers was investigated, taking into account the squared residuals Q vs. Hotelling’s T^2^ plot (Supplementary Materials Figure S7). No sample presented both values outside the 95% of confidence intervals; therefore, all samples were considered in the PLS-DA analysis.

**Figure 8:**
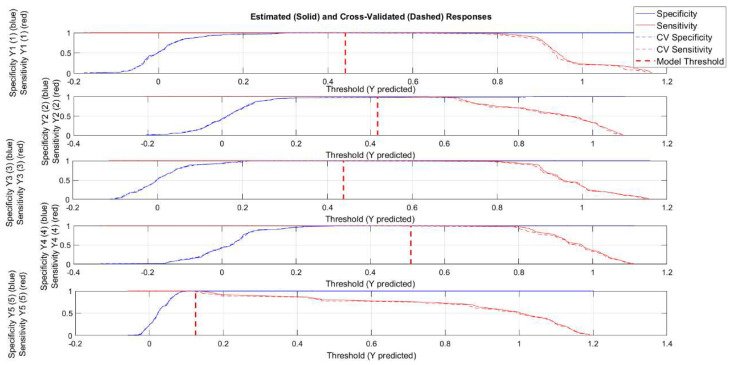
Threshold plots for PLS-DA model. The vertical dashed red line indicates the threshold selected by the PLS-DA algorithm.

However, using a PLS-DA approach on datasets with a limited number of samples, as in this study, could result in overfitting, which means obtaining over-optimistic models. Therefore, a two-method strategy was adopted to avoid this risk. In particular, the Receiver Operating Characteristic (ROC) curve was used to have an overall picture of the classification performance and, together with permutation tests, was carried out to be sure to obtain trustworthy results [55]. Regarding the ROC curve, the area under the curve (AUC) was taken into account, since this value measures the overall method performance. As far as the threshold is concerned, the used PLS-DA algorithm estimated it using Bayes’ Theorem and the available data in order to minimize total errors. Figure 8 shows the sensitivity and specificity as the threshold value is varied. Ideally, these lines cross while still at a value of 1. Crossing below a value of 1 indicates that, as the threshold is increased, sensitivity begins to suffer (drops below 1) before the model is completely specific. The vertical dashed red line indicates the threshold selected by the PLSDA algorithm, which is used to calculate the sensitivity and specificity reported in Table 3. In particular, the point that the vertical threshold line crosses the solid red line in any given plot is the calibration sensitivity, and the point where the threshold line crosses the dashed red line is the cross-validation sensitivity. Similarly, the point where the threshold line crosses the solid blue line is the calibration specificity, and the point where the threshold line crosses the dashed blue line is the cross-validation specificity.

On the other hand, an iterative permutation test involves a random distribution of y-block assigning, at each sample, an incorrect class. Afterwards, different classification models are calculated, and if the performance of these models are significantly and systematically lower than those obtained with the original one, the original model can be assumed to be robust.

Finally, since the sensitivity and specificity values equal to 100% could increase the probability of overfitting, a classification model was also built with a lower number of LVs. In particular, for the whole EOs dataset, 5 LVs were considered, as they presented the second best compromise in terms of classification error average both in calibration and cross-validation (Supplementary Materials Figure S8). The obtained ‘reduced’ model allowed the obtaining of a Sensitivity and Specificity value around 100% in both calibration and cross validation (Figure S9). Therefore, for the final model, 6 LVs were finally chosen according to the criteria previously explained.

## 4. Conclusions

In the present work, ATR-FTIR spectroscopy was demonstrated to efficiently highlight the differences in the chemical composition of EOs belonging to valuable and extremely different genus and species. The PLS-DA models, built on the spectral dataset processed through the first derivative and mean centering, showed excellent predictive performances in terms of specificity and sensitivity, correctly classifying the majority of EOs. The misclassified EOs belonged to *Lavandula* and *Cymbopogon* genera due to the almost identical chemical compositions of some species. The AUC values were higher than 0.92, suggesting the robustness of the classification models. Due to the small number of thyme EOs, the PLS-DA models were not performed, while in the case of rosemary EOs, the models were constructed by combining two different chemotypes. However, preliminary unsupervised PCA was carried out on each dataset, depicting the capability of multivariate statistical tools to differentiate rosemary and thyme EOs, depending on their chemotype (Figures S1 and S2). In conclusion, ATR-FTIR spectroscopy proved to be a promising analytical method for routine analyses of EOs in combination with chemometric tools. By increasing the spectral library of EOs, the here-presented strategy might substitute the conventional and expensive chromatographic method for analyzing all the types of EOs. Additionally, by setting proper confidential levels, the conformity of the EOs to specific quality standards can be assessed.

## Figures and Tables

**Table 1:** Results of PLS-DA models applied on *Mentha*, dataset for both cross-validation of the calibration set and prediction of test set using different pretreatment methods.

*Mentha* Models	Pretreatment	LVs *	Permutation Test in CV	AUC	RMSEC	RMSECV **	RMSEP ***	Sensitivity CV	Specificity CV	Sensitivity Prediction	Specificity Prediction
*M. arvensins* (MA)	Mean centering (MC)	5	0.005	0.95	0.22	0.26	0.30	100	93	100	86
	SNV + MC	5	0.005	0.95	0.20	0.24	0.29	100	93	100	86
	MSC + MC	4	0.005	0.96	0.24	0.29	0.31	89	93	100	93
	1st derivative + MC	3	0.005	0.96	0.23	0.25	0.27	100	93	100	86
	2nd derivative + MC	3	0.005	0.96	0.21	0.24	0.27	100	93	100	86
*M. piperita* (MP)	Mean centering (MC)	5	0.005	0.93	0.22	0.25	0.30	88	97	75	94
	SNV + MC	5	0.005	0.93	0.20	0.24	0.29	87	97	75	81
	MSC + MC	4	0.005	0.92	0.25	0.30	0.31	88	94	75	100
	1st derivative + MC	3	0.005	0.94	0.23	0.25	0.27	88	97	88	100
	2nd derivative + MC	3	0.005	0.96	0.21	0.23	0.28	88	97	88	100
*M. spicata* (MS)	Mean centering (MC)	5	0.005	1	0.03	0.03	0.02	100	100	100	100
	SNV + MC	5	0.005	1	0.02	0.02	0.02	100	100	100	100
	MSC + MC	5	0.005	1	0.06	0.08	0.08	100	100	100	100
	1st derivative + MC	3	0.005	1	0.03	0.03	0.02	100	100	100	100
	2nd derivative + MC	3	0.005	1	0.03	0.03	0.03	100	100	100	100

**Table 2:** Results of PLS-DA models applied on *S. Rosmarinus* dataset for both cross-validation of the calibration set and prediction of test set using different pretreatment methods.

*S. Rosmarinus* Models	Pretreatment	LVs *	Permutation Test in CV	AUC	RMSEC **	RMSECV ***	RMSEP ****	Sensitivity CV	Specificity CV	Sensitivity Prediction	Specificity Prediction
Camphor	Mean centering (MC)	3	0.005	1	0.18	0.20	0.16	100	100	100	100
	SNV + MC	2	0.005	1	0.22	0.23	0.17	100	97	100	100
	MSC + MC	3	0.005	1	0.23	0.25	0.18	100	90	100	100
	1st derivative + MC	2	0.005	1	0.18	0.19	0.16	100	100	100	100
	2nd derivative + MC	2	0.005	1	0.18	0.19	0.16	100	100	100	100
Cineole	Mean centering (MC)	3	0.005	1	0.12	0.13	0.08	100	100	100	100
	SNV + MC	2	0.005	1	0.14	0.14	0.10	100	100	100	100
	MSC + MC	3	0.005	1	0.15	0.17	0.11	100	100	100	100
	1st derivative + MC	2	0.005	1	0.12	0.13	0.10	100	100	100	100
	2nd derivative + MC	2	0.005	1	0.12	0.12	0.09	100	100	100	100
Others	Mean centering (MC)	3	0.005	1	0.16	0.18	0.17	100	100	100	100
	SNV + MC	2	0.005	1	0.23	0.25	0.19	100	97	100	100
	MSC + MC	3	0.005	1	0.21	0.24	0.19	100	100	100	100
	1st derivative + MC	2	0.005	1	0.18	0.19	0.17	100	100	100	100
	2nd derivative + MC	2	0.005	1	0.17	0.18	0.17	100	100	100	100

**Table 3:** Results of PLS-DA models applied on *Cymbopogon* dataset for both cross-validation of the calibration set and prediction of test set using different pretreatment methods.

*Cymbopogon* Models	Pretreatment	LVs *	Permutation Test in CV	AUC	RMSEC **	RMSECV ***	RMSEP ****	Sensitivity CV	Specificity CV	Sensitivity Prediction	Specificity Prediction
*C. citratus* (CC)	Mean centering (MC)	3	0.005	1	0.04	0.04	0.09	100	100	100	100
	SNV + MC	3	0.005	1	0.03	0.04	0.08	100	100	100	100
	MSC + MC	3	0.005	1	0.04	0.04	0.09	100	100	100	100
	1st derivative + MC	3	0.005	1	0.03	0.04	0.09	100	100	100	100
	2nd derivative + MC	3	0.005	1	0.03	0.04	0.08	100	100	100	100
*C. martinii* (CM)	Mean centering (MC)	3	0.005	1	0.14	0.15	0.14	100	100	100	100
	SNV + MC	3	0.005	1	0.14	0.15	0.14	100	100	100	100
	MSC + MC	3	0.005	1	0.15	0.16	0.16	100	100	100	100
	1st derivative + MC	3	0.005	1	0.14	0.15	0.15	100	100	100	100
	2nd derivative + MC	3	0.005	1	0.13	0.15	0.17	100	100	100	100
*C. nardus* (CN)	Mean centering (MC)	3	0.008	0.92	0.31	0.34	0.27	79	91	83	94
	SNV + MC	3	0.01	0.98	0.30	0.33	0.26	86	91	83	94
	MSC + MC	3	0.02	0.91	0.31	0.35	0.26	79	88	100	94
	1st derivative + MC	3	0.008	0.93	0.29	0.32	0.28	86	94	83	89
	2nd derivative + MC	3	0.006	0.94	0.29	0.33	0.28	86	94	83	89
*C. winterianus* (CW)	Mean centering (MC)	3	0.04	0.91	0.29	0.33	0.34	80	86	67	94
	SNV + MC	3	0.02	0.93	0.28	0.31	0.31	80	89	83	89
	MSC + MC	3	0.03	0.90	0.29	0.33	0.30	80	94	83	94
	1st derivative + MC	3	0.02	0.93	0.28	0.31	0.34	80	89	67	89
	2nd derivative + MC	3	0.02	0.92	0.27	0.31	0.33	80	89	67	89

**Table 4:** Results of PLS-DA models applied on *Lavandula* dataset for both cross-validation of the calibration set and prediction of test set using different pretreatment methods.

*Lavandula* Models	Pretreatment	LVs *	Permutation Test in CV	AUC	RMSEC **	RMSECV ***	RMSEP ****	Sensitivity CV	Specificity CV	Sensitivity Prediction	Specificity Prediction
*L. angustifolia* (LA)	Mean centering (MC)	5	0.006	0.93	0.25	0.30	0.29	88	96	100	93
	SNV + MC	6	0.005	1	0.15	0.23	0.38	100	100	88	93
	MSC + MC	5	0.006	0.93	0.25	0.30	0.29	88	91	100	86
	1st derivative + MC	4	0.005	0.95	0.25	0.30	0.28	88	96	100	79
	2nd derivative + MC	4	0.005	0.97	0.24	0.30	0.26	88	96	100	86
*L. latifolia* (LL)	Mean centering (MC)	5	0.005	1	0.08	0.11	0.13	100	100	100	100
	SNV + MC	6	0.005	1	0.07	0.10	0.08	100	100	100	100
	MSC + MC	5	0.005	1	0.08	0.11	0.15	100	100	100	100
	1st derivative + MC	4	0.005	1	0.06	0.08	0.10	100	100	100	100
	2nd derivative + MC	4	0.005	1	0.07	0.09	0.10	100	100	100	100
*L. x intermedia* (LI)	Mean centering (MC)	5	0.006	0.94	0.24	0.30	0.36	93	92	88	100
	SNV + MC	6	0.005	0.99	0.16	0.25	0.38	100	96	88	93
	MSC + MC	5	0.007	0.93	0.26	0.31	0.37	93	92	75	100
	1st derivative + MC	4	0.008	0.95	0.25	0.30	0.34	100	92	88	100
	2nd derivative + MC	4	0.008	0.98	0.23	0.29	0.33	93	92	75	100

* Latent Variables number; ** Root mean square error in calibration; *** Root mean squares error in cross-validation; **** Root mean squares error in prediction.

**Table 5:** Number of samples in the calibration and external validation set. The last raw reports the total number N of samples per class.

PLS-DA First Derivative + MC 6 LVs	*Cymbopogon*	*Lavandula*	*Mentha*	*Rosmarinus*	*Thymus vulgaris*
Calibration set	46	38	48	50	36
External validation set	24	22	24	26	24
Total samples	70	60	72	76	60

**Table 6:** PLS-DA results for the whole EOs dataset.

PLS-DA First Derivative + MC 6 LVs	*Cymbopogon*	*Lavandula*	*Mentha*	*Rosmarinus*	*Thymus vulgaris*
R^2^_CV_ *	96%	86%	90%	94%	84%
RMSEC **	0.08	0.14	0.08	0.10	0.15
RMSECV ***	0.09	0.14	0.08	0.10	0.15
RMSEP ****	0.10	0.16	0.10	0.10	0.23
Permutation test in CV (Rand *t*-test)	0.005	0.005	0.005	0.005	0.005
AUC CV	1	1	1	1	1
Sensitivity CV (%)	100	100	100	100	100
Specificity CV (%)	100	98	100	100	100
Sensitivity in pred (%)	100	100	100	100	100
Specificity in pred (%)	100	96	100	100	100

* Explained variance in cross-validation. ** Root mean squares error in calibration. *** Root mean squares error in cross-validation. **** Root mean squares error in prediction.

## Data Availability

The data presented in this study are available on request from the corresponding author.

## Supplementary Materials

The following supporting information can be downloaded at: https://www.mdpi.com/article/10.3390/molecules27175618/s1. Table S1. Chemical percent composition of one representative sample for each Mentha species: arvensis (MA), piperita (MP), and spicata (MS). Table S2. Chemical percent composition of one representative sample for each chemotype of Salvia rosmarinus. Table S3. Chemical percent composition of one representative sample for each species of Cymbopogon genus. Table S4. Chemical percent composition of one representative sample for each species of Lavandula genus. Table S5. Chemical percent composition of one representative sample for each chemotype of Thymus vulgaris. Figure S1. Results of PCA on ATR-FTIR spectral data of *T. vulgaris* essential oils. Score plots of PC1/PC2 and PC1/PC3, and loading plots of PC1, PC2, and PC3. Figure S2. Results of PCA on ATR-FTIR spectral data of *S. rosmarinus* essential oils. Score plots of PC1/PC2 and loading plots of PC1 and PC2. Figure S3. Variable importance on projection (VIP) score plots for the PLS-DA models for discriminating *Mentha* essential oils. Figure S4. Variable importance on projection (VIP) score plots for the PLS-DA models for discriminating *S. Rosmarinus* essential oils. Figure S5. Variable importance on projection (VIP) score plots for the PLS-DA models for discriminating *Cymbopogon* genus essential oils. Figure S6. Variable importance on projection (VIP) score plots for the PLS-DA models for discriminating *Lavandula* genus essential oils. Figure S7. Squared residuals Q vs. Hotelling’s T2 plot, obtained for PLS-DA model built with 6LVs on the whole EOs dataset. The dashed horizontal and vertical lines show the amplitude of the 95% confidence interval for both parameters. Samples are marked according to their class. Figure S8. Classification Error Average of PLS-DA models built on the whole EOs dataset vs. Latent Variables Number. Figure S9. Threshold plots for PLS-DA model built with 5LVs on the whole EOs dataset. The vertical dashed red line indicates the threshold selected by the PLS-DA algorithm.

## Sample Availability

Samples of the compounds are available from the authors.
