# Supplementary material for: Rapid Classification and Recognition Method of the Species and Chemotypes of Essential Oils by ATR-FTIR Spectroscopy Coupled with Chemometrics
Source: Molecules. 2022 Aug 31;27(17):5618. doi: 10.3390/molecules27175618 (PMC9458032; doi:10.3390/molecules27175618)
Supplement: Supplementary file 1 [file molecules-27-05618-s001.zip › molecules-1816355-supplementary.pdf]

Table S1. Chemical percent composition of one representative sample for each *Mentha* species: *arvensis* (MA), *piperita* (MP), and *spicata* (MS).

	LRI	*Arvensis* (MA)	*Arvensis* menthol-rich (MA)	*Piperita* (MP)	Spicata (MS)
α-pinene	930	0.104	0.638	1.170	1.110
sabinene	970	0.204	0.278	/	0.446
β-pinene	973	/	0.740	1.951	1.030
myrcene	990	0.143	0.559	0.204	2.029
3-octanol	995	/	0.379	0.672	0.172
o-cymene	1022	/	/	0.508	0.166
limonene	1027	1.701	3.018	4.596	24.863
1,8-cineole	1030	/	/	3.368	/
γ-terpinene	1057	/	/	0.164	/
linalool	1100	0.160	0.287	0.129	/
isopulegol	1147	2.117	0.823	0.111	0.578
menthone	1158	22.718	6.538	23.875	0.404
isomenthone	1168	20.077	6.038	13.408	1.266
menthol	1183	42.435	74.040	34.656	0.228
p cymen-8-ol	1187	0.887	0.367	0.584	0.201
α-terpineol	1191	0.553	0.218	0.532	1.936
nerol	1228	/	/	/	0.195
pulegone	1243	1.568	0.664	1.656	0.216
carvone	1258	1.755	0.428	0.590	57.772
geranial	1276	0.398	/	0.456	1.042
menthyl acetate	1296	1.179	2.816	6.206	0.984
geranyl acetate	1388	0.358	0.118	0.159	0.108
β-elemene	1394	0.129	/	/	0.979
β-caryophyllene	1423	0.295	0.402	1.498	0.297
α-humulene	1459	0.194	/	0.186	1.129
ar curcumene	1487	0.183	0.275	0.328	0.230
viridiflorene	1503	0.545	/	0.382	0.155
Total		97.700	98.630	97.390	97.390

Table S2. Chemical percent composition of one representative sample for each chemotype of *Salvia rosmarinus*.

	LRI	Pinene	Verbenone	Cineole	Camphor
unknown	918	0.461	/	0.167	0.304
α-thujene	924	0.123	0.422	0.44	0.111
α-pinene	934	46.169	19.421	11.631	25.96
camphene	946	9.278	5.721	4.93	10.829
benzaldehyde	950	1.154	1.235	/	0.183
sabinene	973	2.613	1.853	9.29	3.587
β-pinene	976	0.145	/	0.101	0.125
3-octanone	988	2.667	/	/	0.868
myrcene	992	5.095	1.552	1.467	2.305
α-phellandrene	1004	0.325	0.335	0.178	0.113
3-carene	1009	/	/	0.267	0.148
α-terpinene	1014	0.608	0.506	0.415	/
o-cymene	1022	0.510	1.257	1.181	2.227
1,8-cineole	1029	10.58	11.585	47.744	21.526
γ-terpinene	1056	0.970	1.100	0.792	0.178
trans linalool oxide	1086	1.138	1.289	0.417	0.210
linalool	1100	1.458	1.786	0.714	1.587
camphor	1143	4.007	14.088	9.993	20.26
borneol	1167	2.214	3.581	2.574	2.134
cis-3-pinanone	1173	0.656	1.541	/	0.125
terpinen-4-ol	1177	0.430	0.983	0.713	0.510
α-terpineol	1191	0.467	1.103	1.341	1.575
verbenone	1211	2.202	12.259	/	1.388
carvone	1259	/	0.136	/	/
bornyl acetate	1289	1.838	10.497	0.946	0.55
β-caryophyllene	1422	2.212	/	2.774	0.928
α-humulene	1458	0.296	0.196	0.295	0.33
Total		97.646	92.506	98.430	98.091

Table S3. Chemical percent composition of one representative sample for each species of *Cymbopogon* genus.

	LRI	*Martinii* (CM)	*Citratus* (CC)	*Nardus* (CN)	*Winterianus* (CW)
α-thujene	920	/	0.197	/	/
α-pinene	931	/	0.292	0.115	/
camphene	946	/	1.628	/	/
3-octanone	987	/	1.68	/	/
myrcene	990	0.445	0.173	0.131	0.38
limonene	1026	0.474	0.412	6.947	3.595
cis ocimene	1036	0.456	0.453	/	0.588
trans ocimene	1047	2.023	0.284	/	0.369
cis linalool oxide	1073	/	0.97	/	
linalool	1100	2.919	1.279	0.491	0.808
trans pinocarveol	1139	/	0.121	/	/
camphor	1146	/	0.356	1.003	1.468
citronellal	1155	/	0.252	40.206	39.155
borneol	1167	/	1.092	/	/
terpinen-4-ol	1178	/	0.272	/	0.135
p cymen-8-ol	1185	/	1.302	/	/
α-terpineol	1192	/	0.189	/	/
citronellol	1232	0.137	0.109	12.764	10.994
pulegone	1244	0.416	/	1.787	0.436
neral	1253	/	31.855	/	/
geraniol	1265	82.3	7.134	22.303	21.529
geranial	1277	0.891	40.26	2.543	0.653
geraniol formate	1303	/	/	/	/
citronellyl acetate	1357	/	/	1.698	2.237
geranyl acetate	1388	6.392	3.262	2.854	2.596
β-elemene	1394	/	/	0.623	1.569
β-caryophyllene	1424	0.981	1.513	0.356	0.592
α-humulene	1454	/	0.439	/	/
β-farnesene	1461	/	0.18	/	0.182
germacrene D	1488	/	0.231	0.348	1.368
α-selinene	1499	/	/	0.117	1.087
α-muurolene	1504	/	/	0.178	0.433
γ-cadinene	1522	/	0.998	0.37	0.61

δ-cadinene	1530	/	0.205	0.771	1.403
elemol	1556	/	/	1.355	2.452
caryophyllene oxide	1593	0.132	0.229	/	/
cubenol	1640	/	/	0.103	0.137
Total		97.566	97.367	97.063	94.776

Table S4. Chemical percent composition of one representative sample for each species of *Lavandula* genus.

	LRI	*Angustifolia* (LA)	*x intermedia* (LI)	*Latifolia* (LL)
α-thujene	923	0.239	/	/
α-pinene	930	/	0.526	2.14
camphene	944	0.196	0.340	0.282
sabinene	972	/	0.177	2.703
β-pinene	976	0.330	0.576	/
Oct-1-en-3-ol	984	0.704	/	/
myrcene	989	0.777	1.438	0.517
α-terpinene	1012	0.288	0.162	0.115
o-cymene	1022	0.244	/	0.340
limonene	1026	0.327	1.032	/
1,8-cineole	1028	0.85	5.527	28.98
cis-ocimene	1037	2.808	1.4685	0.238
trans-ocimene	1047	2.869	0.753	0.118
γ-terpinene	1056	/	0.153	0.295
cis linalool oxide	1070	0.27	0.132	0.173
trans linalool oxide	1086	0.229	0.464	0.306
linalool	1105	29.958	30.812	40.419
fenchol	1113	1.142	0.521	/
camphor	1142	0.495	6.956	10.834
borneol	1164	0.951	3.0185	1.312
lavandulol	1168	0.928	0.448	/
terpinen-4-ol	1178	2.723	2.077	0.339
p cymen-8-ol	1185	0.175	/	0.129
α-terpineol	1190	0.371	0.817	1.110
myrtenal	1193	0.324	0.194	0.144
thymol methyl ether	1241	/	0.000	0.203
pulegone	1246	/	0.109	0.144
linalyl acetate	1265	38.309	30.165	0.698

lavandulyl acetate	1293	3.685	2.927	/
neryl acetate	1366	0.242	0.331	/
β-cubebene	1385	0.538	0.703	0.174
β-caryophyllene	1424	4.537	1.874	1.535
α-humulene	1458	1.503	1.374	0.510
ar curcumene	1485	0.291	0.966	0.537
γ-cadinene	1518	0.152	0.397	0.199
α-calacorene	1547	/	/	2.012
caryophyllene oxide	1591	0.732	/	0.173
Total		97.187	96.434	96.678

Table S5. Chemical percent composition of one representative sample for each chemotype of *Thymus vulgaris.*

	LRI	Thymol	Cymene-thymol	Linalool	Borneol
Unknown	919	0.105	/	/	0.426
α-thujene	924	1.042	/	0.189	0.440
α-pinene	931	1.126	3.099	8.279	4.993
camphene	945	1.086	1.010	0.828	8.554
sabinene	973	0.222	0.384	0.817	0.956
β-pinene	978	/	/	0.469	/
3-octanone	987	/	0.109	/	/
myrcene	990	2.059	1.156	4.393	0.858
α-phellandrene	1003	0.241	/	0.177	0.120
α-terpinene	1015	1.759	/	1.316	0.625
o-cymene	1023	/	/	/	3.650
p-cymene	1026	19.319	40.063	5.740	1.207
limonene	1028	0.764	0.864	3.055	0.614
1,8-cineole	1031	/	0.559	/	/
trans ocimene	1047	/	/	/	/
γ-terpinene	1057	10.218	0.210	5.343	1.620
cis inalool oxide	1069	0.223	/	0.477	/
trans linalool oxide	1088	0.234	/	1.542	0.383
linalool	1101	4.541	5.461	47.151	3.981
camphor	1144	0.598	0.898	0.183	2.010
borneol	1166	1.519	1.097	1.315	30.968
terpinen-4-ol	1177	1.234	0.308	10.102	1.741

p cymen-8-ol	1186	0.167	/	0.426	/
α-terpineol	1190	0.210	9.434	1.406	9.549
myrtenal	1197	/	1.371	0.358	0.315
nerol	1229	/	/	0.198	0.392
carvone	1249	0.222	/	0.119	3.206
linalyl acetate	1260	/	/	0.561	0.112
bornyl acetate	1287	/	/	0.193	1.849
Thymol	1294	46.298	31.066	0.529	7.763
carvacrol	1304	4.241	/	0.134	4.562
β-cubebene	1386	/	/	/	/
β-caryophyllene	1424	1.514	0.920	0.582	4.393
α-humulene	1459	/	/	/	0.228
alloaromadendrene	1466	/	/	/	0.122
ar curcumene	1486	/	/	/	0.122
viridiflorene	1501	/	/	/	0.100
γ-cadinene	1519	/	/	/	0.308
δ-cadinene	1529	/	/	/	0.497
elemol	1555	/	/	/	
caryophyllene oxide	1591	0.122	/	0.200	0.247
Total		98.897	98.036	95.823	97.337

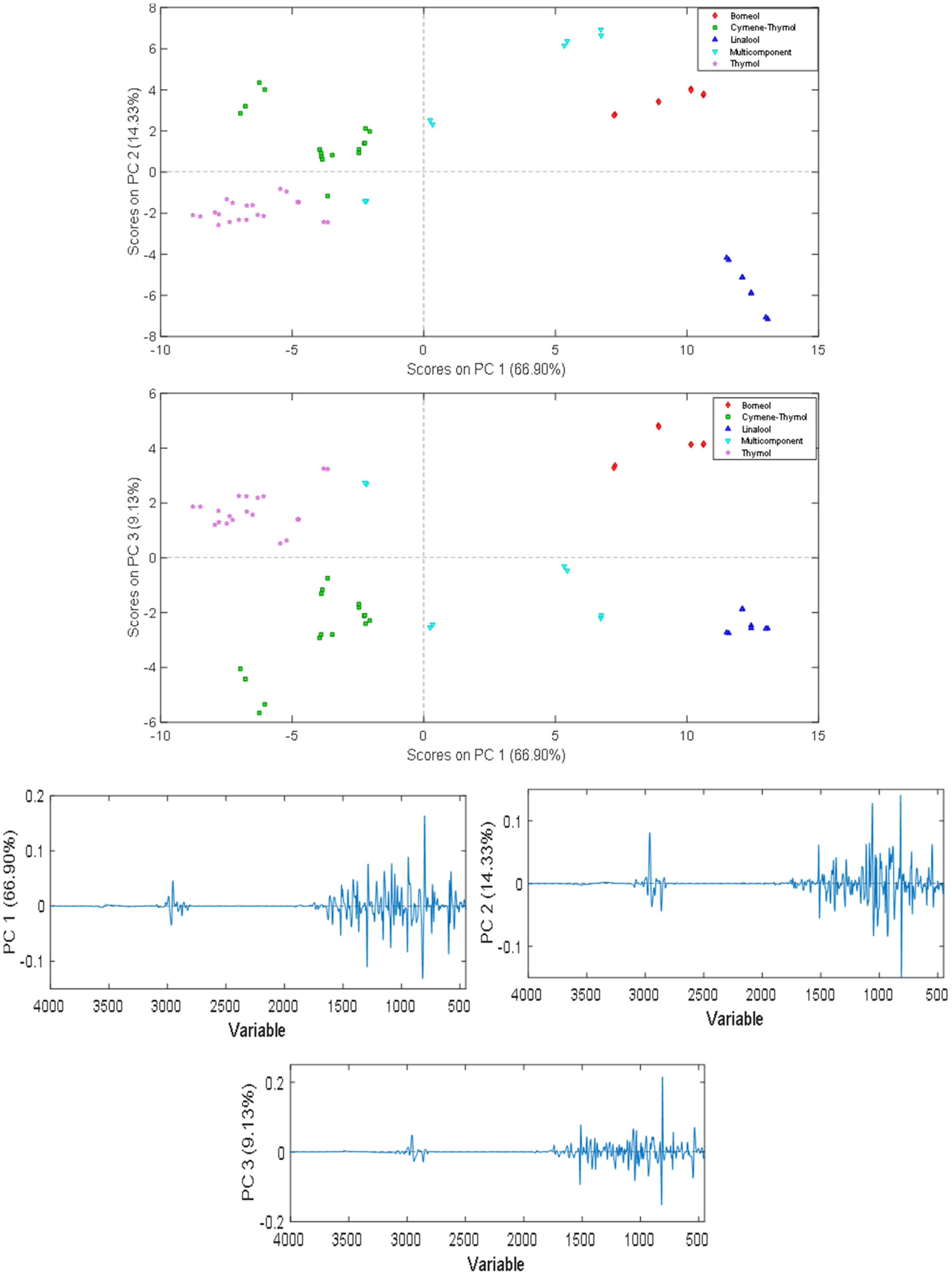

Figure S1. Results of PCA on ATR-FTIR spectral data of *T. vulgaris* essential oils. Score plots of PC1/PC2 and PC1/PC3, and loading plots of PC1, PC2, and PC3.

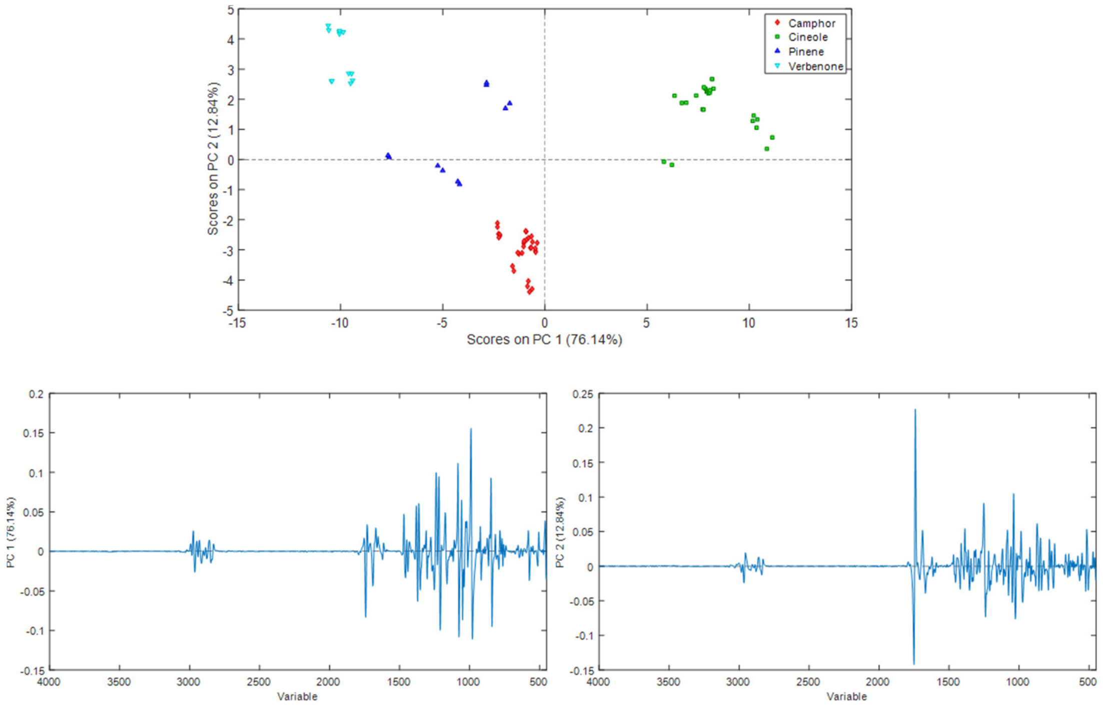

Figure S2. Results of PCA on ATR-FTIR spectral data of *S. rosmarinus* essential oils. Score plots of PC1/PC2 and loading plots of PC1 and PC2.

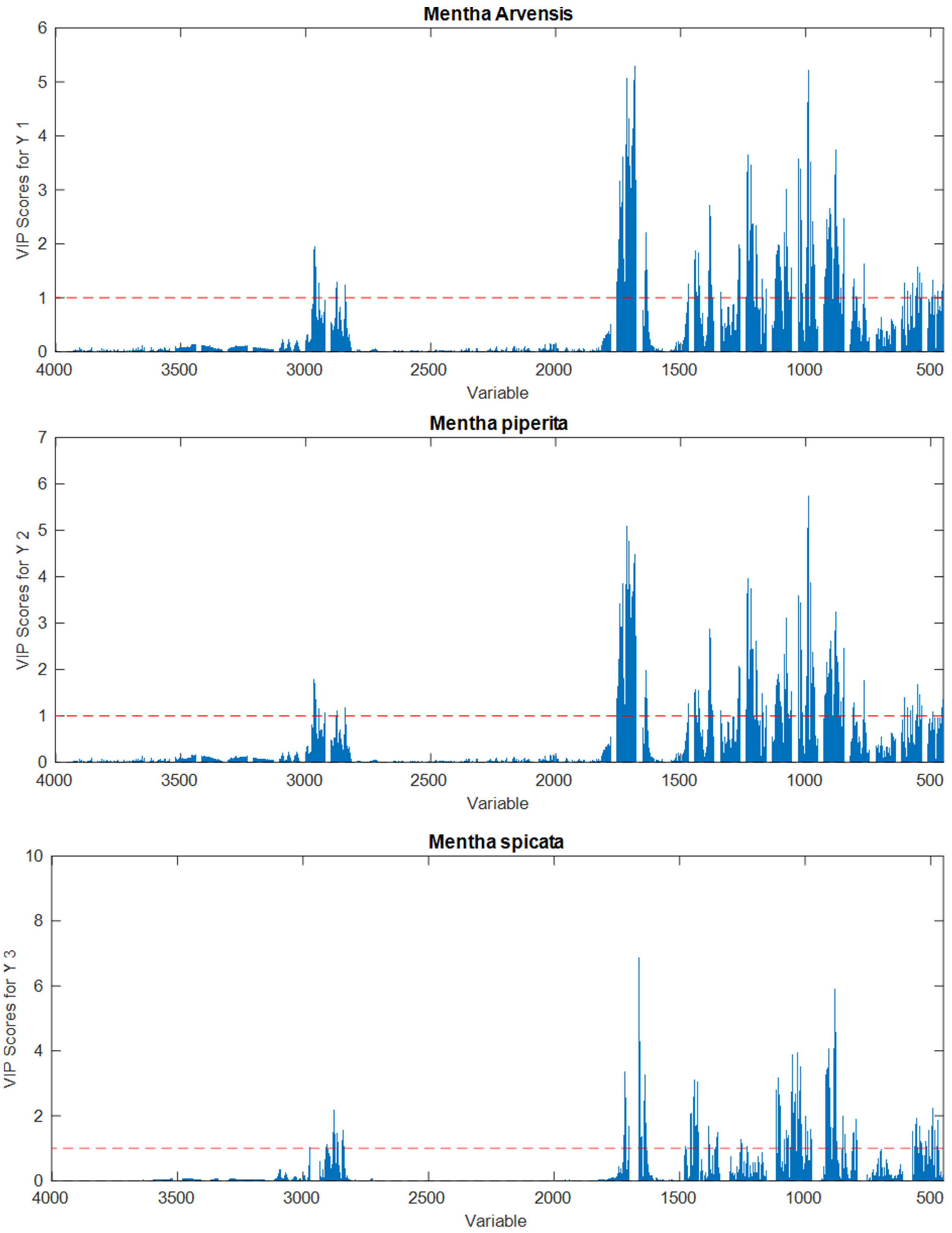

Figure S3. Variable importance on projection (VIP) score plots for the PLS-DA models for discriminating *Mentha* essential oils.

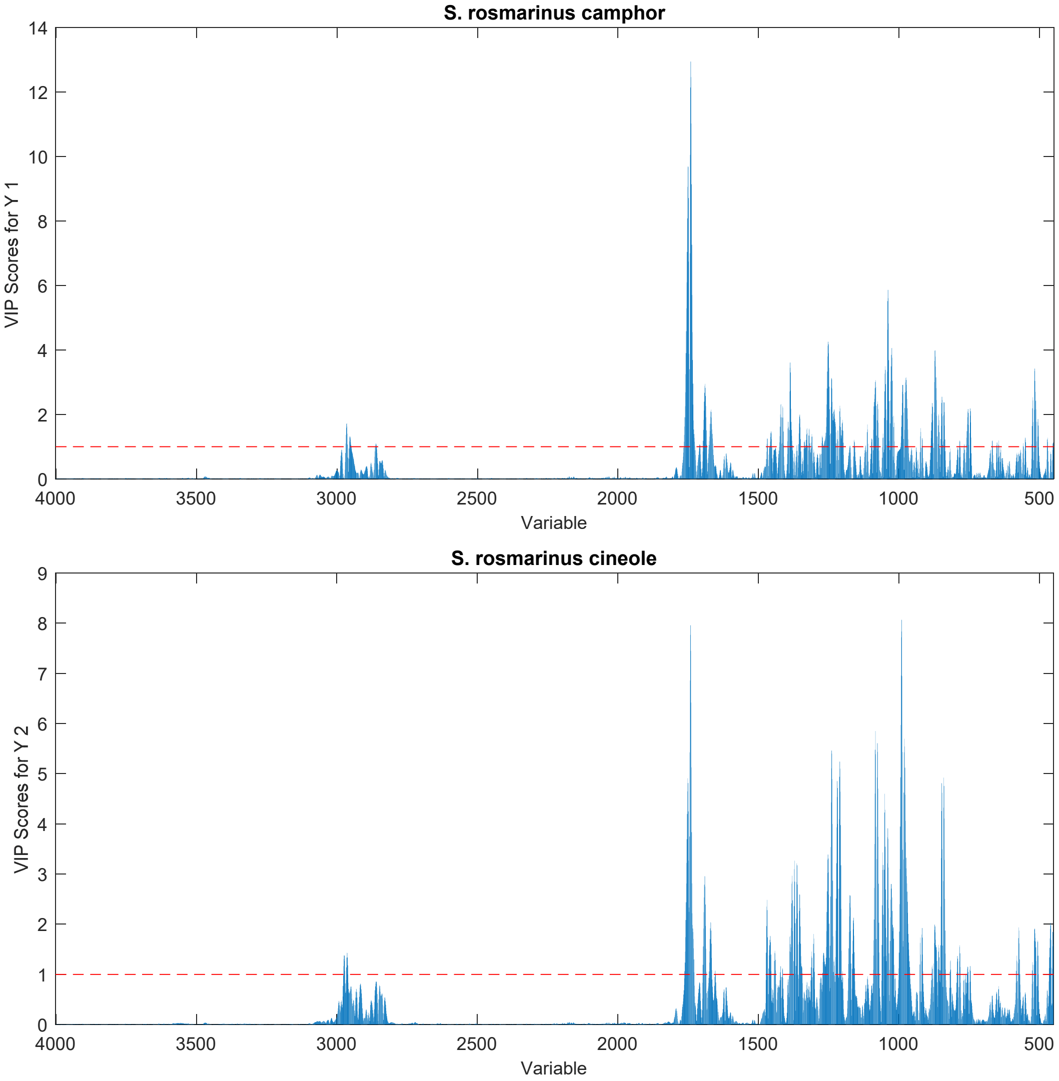

Figure S4. Variable importance on projection (VIP) score plots for the PLS-DA models for discriminating *S. Rosmarinus* essential oils.

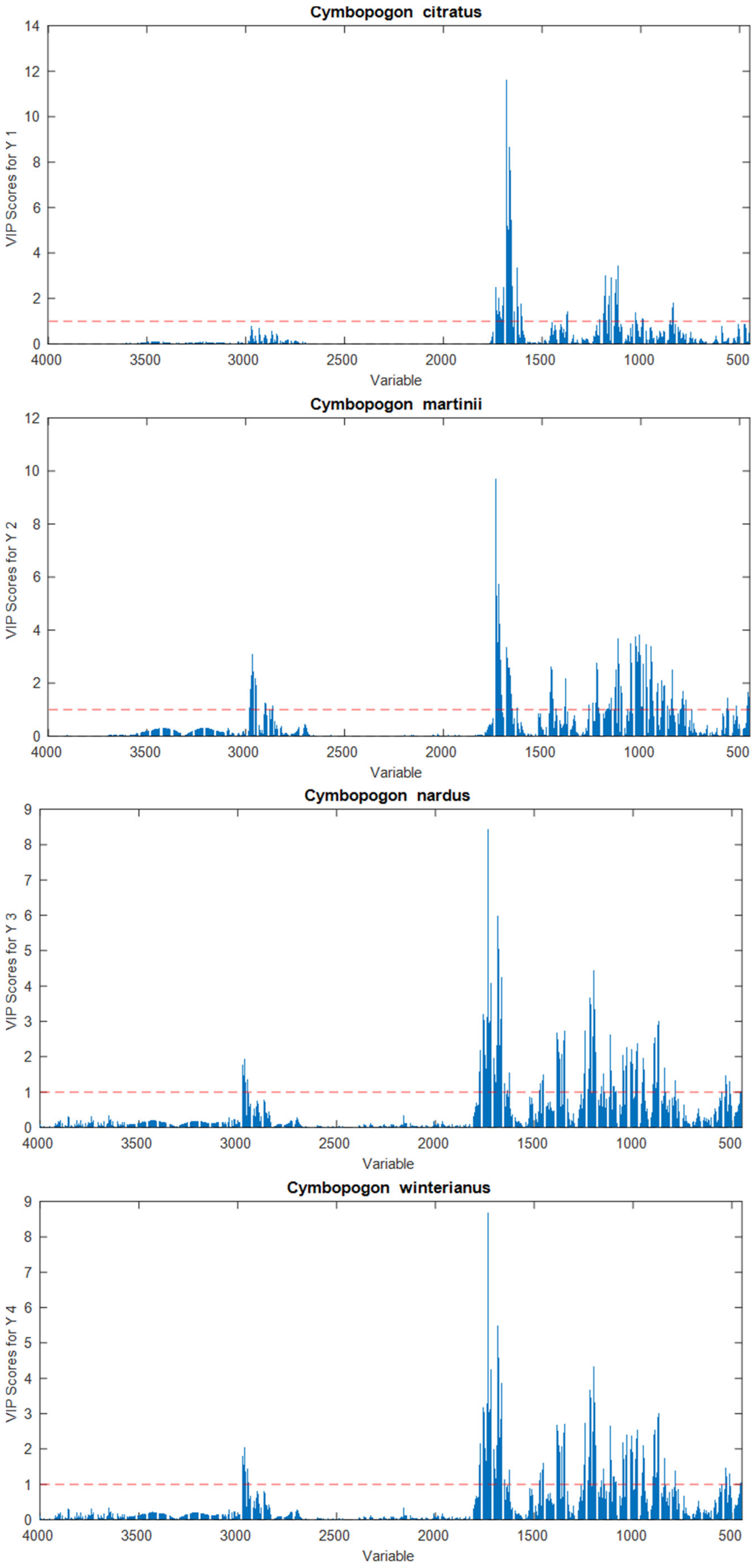

Figure S5. Variable importance on projection (VIP) score plots for the PLS-DA models for discriminating *Cymbopogon* genus essential oils.

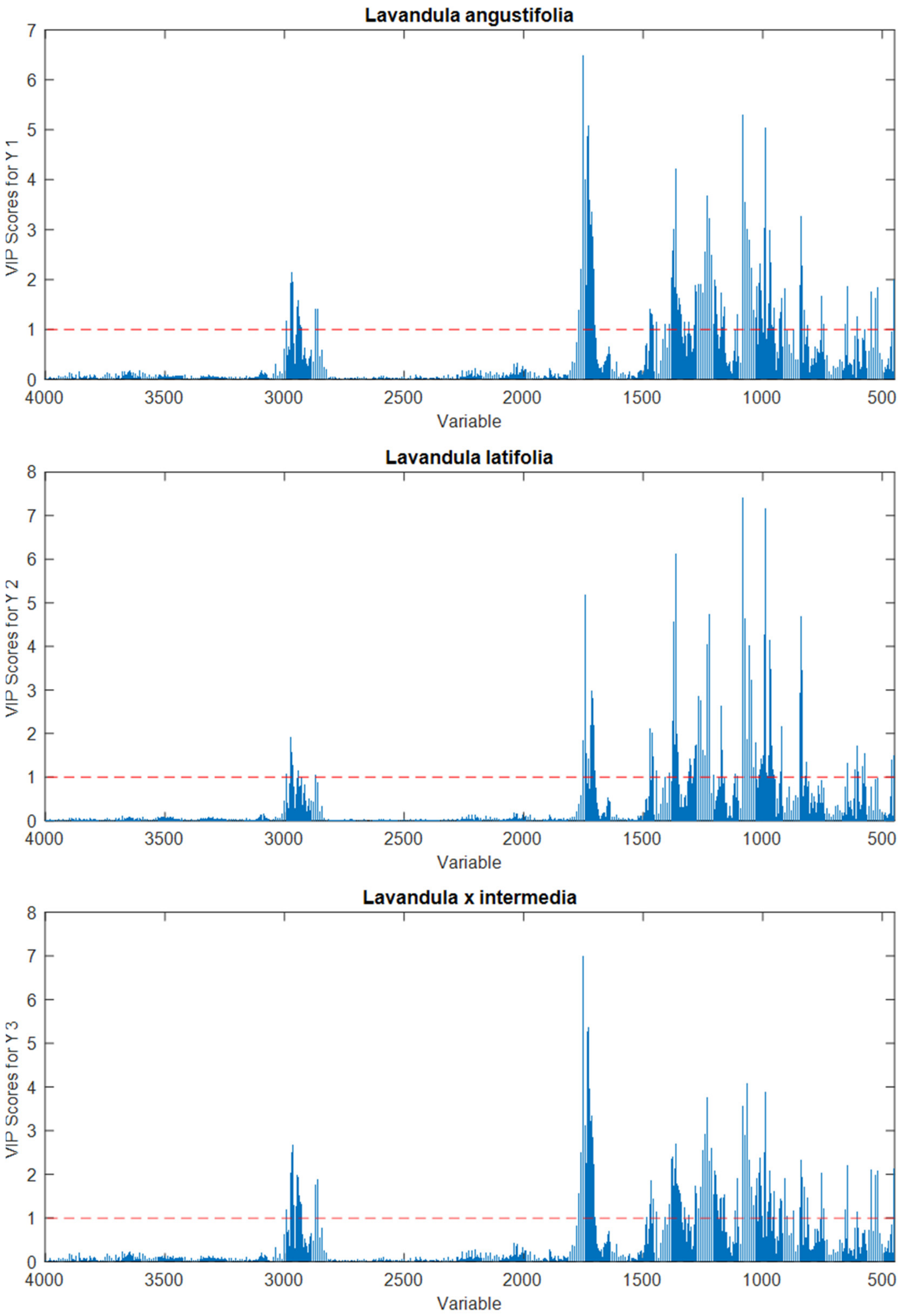

Figure S6. Variable importance on projection (VIP) score plots for the PLS-DA models for discriminating *Lavandula* genus essential oils.

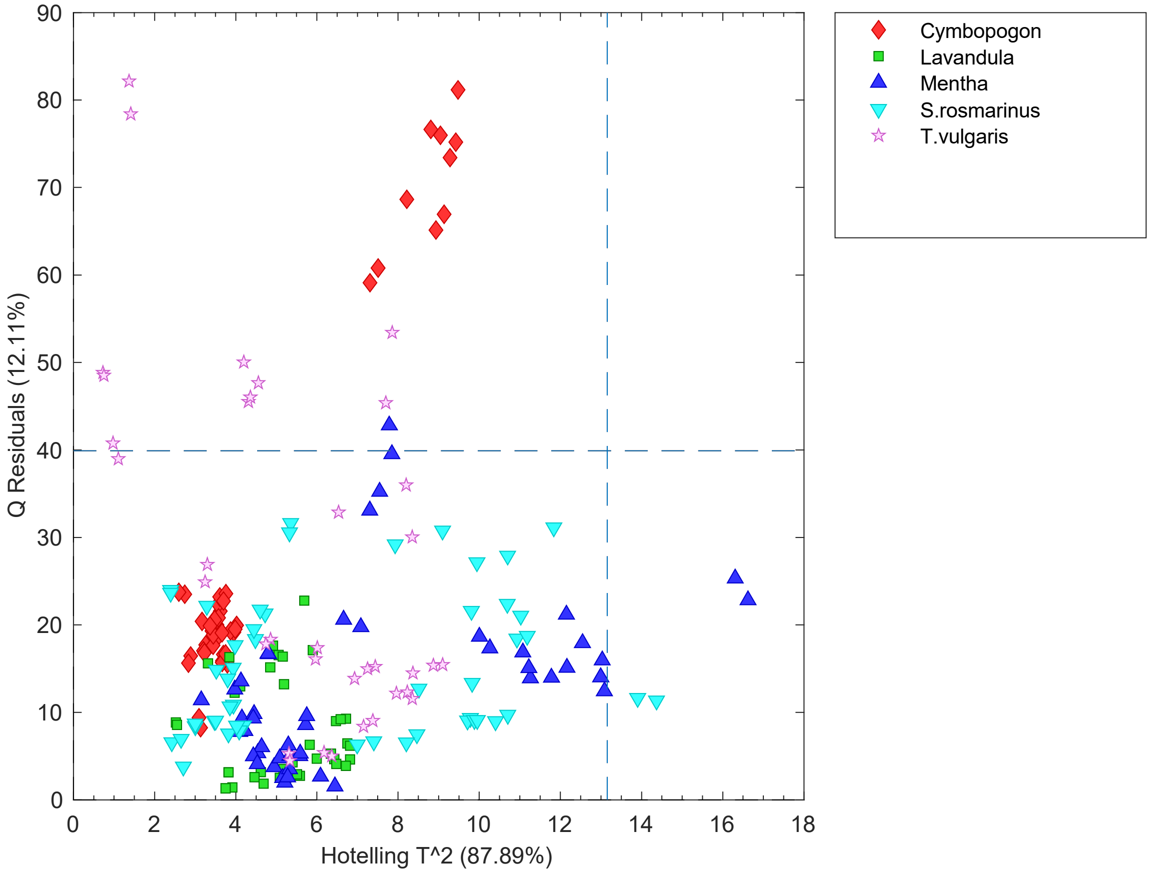

Figure S7. Squared residuals Q vs. Hotelling's T2 plot, obtained for PLS-DA model built with 6LVs on the whole EOs dataset. The dashed horizontal and vertical lines show the amplitude of the 95% confidence interval for both parameters. Samples are marked according to their class.

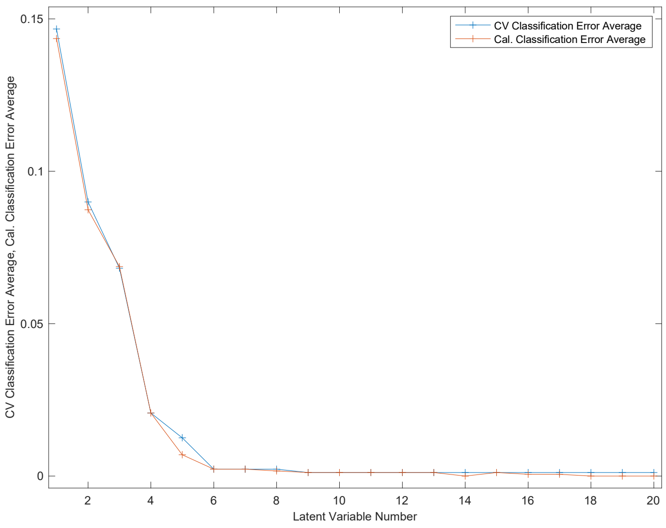

Figure S8. Classification Error Average of PLS-DA models built on the whole EOs dataset vs. Latent Variables Number

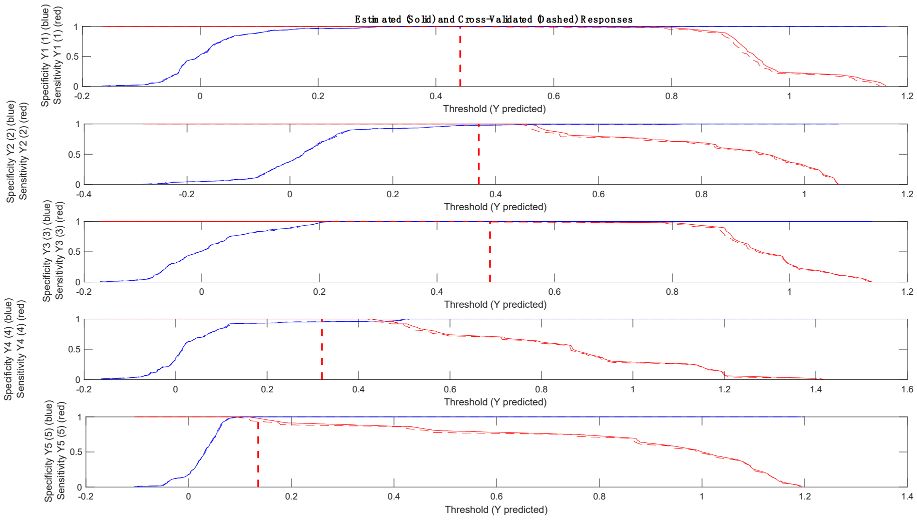

Figure S9. Threshold plots for PLS-DA model built with 5LVs on the whole EOs dataset. The vertical dashed red line indicates the threshold selected by the PLS-DA algorithm.
